# Methods that remove batch effects while retaining group differences may lead to exaggerated confidence in downstream analyses

**DOI:** 10.1093/biostatistics/kxv027

**Published:** 2015-08-13

**Authors:** Vegard Nygaard, Einar Andreas Rødland, Eivind Hovig

**Affiliations:** Department of Tumor Biology, Institute for Cancer Research, Oslo University Hospital HF – Radiumhospitalet, Montebello, 0310 Oslo, Norway; Department of Tumor Biology, Institute for Cancer Research, Oslo University Hospital HF – Radiumhospitalet, Montebello, 0310 Oslo, Norway; Institute of Cancer Genetics and Informatics, Oslo University Hospital HF – Radiumhospitalet, Montebello, 0310 Oslo, Norway and Department of Informatics, University of Oslo, 0316 Oslo, Norway

**Keywords:** Batch effects, Data normalization, Microarrays, Reproducible research

## Abstract

Removal of, or adjustment for, batch effects or center differences is generally required when such effects are present in data. In particular, when preparing microarray gene expression data from multiple cohorts, array platforms, or batches for later analyses, batch effects can have confounding effects, inducing spurious differences between study groups. Many methods and tools exist for removing batch effects from data. However, when study groups are not evenly distributed across batches, actual group differences may induce apparent batch differences, in which case batch adjustments may bias, usually deflate, group differences. Some tools therefore have the option of preserving the difference between study groups, e.g. using a two-way ANOVA model to simultaneously estimate both group and batch effects. Unfortunately, this approach may systematically induce incorrect group differences in downstream analyses when groups are distributed between the batches in an unbalanced manner. The scientific community seems to be largely unaware of how this approach may lead to false discoveries.

## 1. Introduction

Extraneous variables, if left unaccounted for, have the potential to lead an investigator into drawing wrong conclusions. A common example is “batch effects” caused by reagents, microarray chips, and other equipment made in batches that may vary in some way, which often have systematic effects on the measurements. A similar example is “center effects”, when samples or data come from multiple sources. See Luo *and others* (2010) for more examples.

**Fig. 1.**
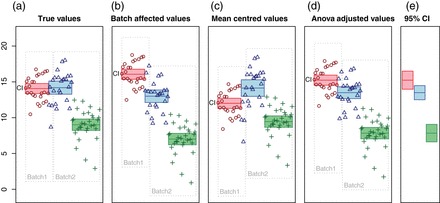
We simulated expression of one gene from three study groups unevenly distributed in two batches, designed to illustrate the spurious effects that may arise from different batch adjustments. The *Y*-axis represents the expression values, while the *X*-axis is used to visually separate the groups and batches. Circles, triangles, and crosses indicate values from each of the three groups. Surrounding boxes indicate batches. For each group, the filled bar indicates mean and its 95% confidence interval, which give some indication as to which group differences would be found significant. (a) The “true values” measured in a system without batch effects. The first two groups are drawn from normal distributions with the same mean and variance, while the third has a lower mean. (b) Batch effects have been added to the “true values”, as indicated by the shifting of the batch frames, leading to apparent differences between the first two groups. (c) The measurements in (b) are adjusted by mean-centering each batch. All groups appear significantly different. (d) The measurements in (b) are adjusted with two-way ANOVA-based batch centering (using limma). Again, all groups appear to be significantly different. (e) The least squares estimates of the group means from a two-way ANOVA have the same means as in (d), but more appropriate confidence intervals.

In a typical experiment comparing differences between study groups, presence of batch effects will decrease statistical power, since it adds variation to the data. If the batch–group design is unbalanced, i.e. if the study groups are not equally represented in all batches, batch effects may also act as a confounder and induce false differences between groups (Leek *and others*, 2010) as illustrated in Figure 1(b).

The standard way to handle an extraneous variable is to include it in the statistical model employed in the inquiry. However, many analysis tools for high throughput data do not cater for this option, and when available, it could still be outside the competence of the investigator. Therefore, an alternative two-step procedure has emerged. First the batch effects are estimated and removed, creating a “batch effect free” data set. Then, the statistical analyses are performed on the adjusted data without further consideration of batch effects. This appealing compartmentalization is also convenient for practical purposes, for example when data-processing and statistical analyses are performed by different personnel. Unfortunately, as we demonstrate in this paper, when the batch–group design is unbalanced, this approach may be unreliable.

A simple removal of batch effects can be achieved by subtracting the mean of the measurements in one batch from all measurements in that batch, i.e zero-centering or one-way ANOVA adjustment as implemented in the method pamr.batchadjust from the pamr package in R. When the batch–group design is balanced, zero-centering will remove most, but not necessarily all, of the variance attributed to batch and leave the between-group variance, thus increasing statistical power. However, when the batch–group design is unbalanced, batch differences will in part be influenced by group differences, and thus batch correction will reduce group differences and thereby reduce statistical power. In very uneven group–batch designs with multiple groups, spurious group differences may even be induced in this way. Figure 1(c) illustrates both of these effects.

To mitigate the above problems, one may simultaneously estimate batch effects and group differences, e.g. using a two-way ANOVA, and only remove the batch differences from the data. Effectively, group differences are estimated based on within-batch comparisons, and applied to the batch-adjusted data. In a balanced group–batch design, estimates of group differences and batch effects are independent, and this approach becomes identical to the above-described zero-centering per batch. However, if the group–batch design is heavily unbalanced, estimation of group differences and batch effects are interdependent, and when applying the estimated group differences across the entire data set, point estimates are used while the estimation errors are ignored. One way to view this is that the original batch effects are removed, but instead a new batch effect is introduced by the batch effect estimation errors. If the original batch effects are larger than their estimation errors, this still represents an improvement over not correcting for batch effects. However, in cases where the original batch effects are small and their estimates inaccurate, it may actually worsen the problem. In any case, subsequent analyses that do not account for batch effects may systematically underestimate the size of estimation errors and exaggerate the confidence in group differences. Figure 1(d) illustrates how confidence intervals are deflated by this batch adjustment method by comparing them to confidence intervals from the original ANOVA in Figure 1(e).

Several tools exist for batch-adjusting gene expression data. Some of these also allow covariates to be included in the batch adjustment: e.g. the commercial software Partek Genomics Suite, the R packages limma (Smyth and Speed, 2003), ber (Giordan, 2013), and ComBat (Johnson *and others*, 2007), which is included in the sva package (Leek *and others*, 2012). Most of these use two-way ANOVA, while ComBat uses an empirical Bayes approach to avoid over-correcting which is critical for use with small batches. While the packages allow study group to be included as a covariate during batch adjustment, this feature has generally not been actively promoted, and both Partek and the relevant method “removeBatchEffect” in limma provide warnings that they are not intended for use prior to linear modeling, although we suspect this warning is not always heeded. With ComBat, however, this feature has been more heavily promoted, and it was through this use of ComBat that we ourselves encountered this problem. We note that the sva package has been updated to address these concerns.

When sample or batch sizes are small, statisticians would most likely take extra precautions. However, batch adjustment using two-way ANOVA or ComBat on unbalanced data sets may be just as harmful for large samples and batch sizes as for small. For example, group comparisons using one-way ANOVA on the batch adjusted data will essentially result in $F$-statistics that are inflated by a fixed factor which depends on the unevenness of the design, rather than the size of the sample or batches. The effect of this may be further exacerbated by running these analyses a large number of times, e.g. on thousands of genes, and using false discovery rate (FDR) to determine significant cases: an approach that is particularly sensitive to inflated false positive rates.

The failure to obtain a “batch effect free” data set when batch-adjusting data where study groups are unevenly distributed across batches has also been observed in Buhule *and others* (2014). Proper statistical analysis of unbalanced designs require models that handle batch effects as part of the analysis. However, the best approach is to ensure a balanced study design from the start, to avoid data analysis problems as well as the loss of statistical power that ensues when batch and group effects need to be disentangled.

## 2. Methods for batch effect correction

### 2.1. Model for data with batch effects

We will base our discussion on a simple, two-way ANOVA model for data with batch effects:

(2.1) \begin{equation*} Y_{ijr}=\alpha+\beta_j+\gamma_i+\epsilon_{ijr} \end{equation*}

where $i=1,\ldots ,m$ are the different batches, $j=1,\ldots ,M$ are different study groups that we wish to compare, and $\epsilon _{ijr}\sim N(0,\sigma ^2)$ are the error terms for samples $r=1,\ldots ,n_{ij}$ within batch $i$ and group $j$.

When combining data from more diverse data sources, e.g. different microarray platforms, a more general model is required. ComBat also uses empirical Bayes estimates across genes to stabilize estimates, which is critical for use with small batches, and will also moderate the side-effects of batch adjustments. However, for cases with large batches or substantial batch effects, this should be similar to the two-way ANOVA approach.

### 2.2. Standard batch correction methods

A common ambition of batch effect adjustments is to remove batch differences in such a way that downstream analyses of the adjusted data may be done without further corrections for batches. We illustrate this using Figure 1, where the first frame contains the “true” values with no batch differences, and the remaining frames show values with various levels of batch effects and batch effect corrections.

#### 2.2.1. Zero-centering batches

The most common method for removing batch effects is to zero-center each batch: $\tilde {Y}^0_{ijr}=Y_{ijr}-\bar {Y}_{i}$ where $\bar {Y}_i=({1}/{n_{i-}}) \sum _{j=1}^M\sum _{r=1}^{n_{ij}} Y_{ijr}$, $n_{i-}=\sum _{j=1}^M n_{ij}$. An alternative is to center each batch to the common average by adding the average value $\bar {Y}$ across the entire data set: i.e. $\tilde {Y}^{{\rm avg}}_{ijr}=\tilde {Y}^0_{ijr}+\bar {Y}$. When comparing groups, the common value $\bar {Y}$ has no effect, and so this mean-centering is equivalent to zero-centering each batch. If the groups are unevenly represented in the different batches, the batch average $\bar {Y}_i$ will tend to capture, through $\bar \beta _i$, group differences, as well as batch effects:

(2.2) \begin{equation*} \tilde{Y}^0_{ijr}=Y_{ijr}-\bar{Y}_{i}=\beta_j-\bar{\beta}_i+\epsilon_{ijr}-\bar{\epsilon}_i \quad{\rm where}\ \bar{\beta}_i={\sum_{j=1}^M \frac{n_{ij}}{n_{i{\rm -}}}\beta_j},\ \bar{\epsilon}_i=\frac{1}{n_{i{\rm -}}} \sum_{j=1}^M\sum_{r=1}^{n_{ij}} \epsilon_{ijr}. \end{equation*}

Thus, batch centering will tend to reduce group differences in an unbalanced design, and reduce the power of downstream analyses. By reducing the differences between some groups, i.e. those found together in the same batches, one may also induce false differences between other groups, as is demonstrated in Figure 1(c).

#### 2.2.2. Batch adjustment using two-way ANOVA to estimate batch effects

Removing batch effects while retaining group differences can be achieved by subtracting the batch effects $\hat \gamma _i$ estimated from the two-way ANOVA model (2.1), yielding $\tilde {Y}^{{\rm cov}}_{ijr}=Y_{ijr}-\hat \gamma _i=\alpha +\beta _j+(\gamma _i-\hat \gamma _i)+\epsilon _{ijr}$. This gives batch-adjusted values, where any systematic bias induced by the batch differences has been removed, while the group differences are retained.

The estimation error $\hat \gamma _i-\gamma _i$ affects all values within the same batch in the same manner. Thus, while the aim is to remove spurious dependencies within batches, it may also induce new dependencies. The batch effect estimation errors will influence group effects in proportion to how well the group is represented in each batch:

(2.3) \begin{equation*} \tilde{Y}^{{\rm cov}}_{{\rm -}j} =\frac{1}{n_{{\rm -}j}} \sum_{i=1}^m\sum_{r=1}^{n_{ij}}\tilde{Y}_{ijr} =\alpha+\beta_j+\bar{\epsilon}_{{\rm -}j} -\sum_{i=1}^m \frac{n_{ij}}{n_{{\rm -}j}}(\hat{\gamma}_i-\gamma_i) \quad{\rm where}\ n_{{\rm -}j}=\sum_{i=1}^m n_{ij} \end{equation*}

so that

(2.4) \begin{equation*} \tilde{Y}^{{\rm cov}}_{{\rm -}j}-\tilde{Y}^{{\rm cov}}_{{\rm -}j'} =(\beta_j-\beta_{j'})+(\bar{\epsilon}_{{\rm -}j}-\bar{\epsilon}_{{\rm -}j'}) -\sum_{i=1}^m \left(\frac{n_{ij}}{n_{{\rm -}j}}-\frac{n_{ij'}}{n_{{\rm -}j'}}\right)(\hat{\gamma}_i-\gamma_i). \end{equation*}

In a balanced group–batch design, the estimation error $\hat \gamma _i-\gamma _i$ has the same effect for all groups, and thus does not influence group comparisons. In an unbalanced design, however, it will induce increased differences between groups which, when ignored in downstream analyses, may lead to over-confidence in estimated group differences. In Figure 1(d), the effect of batch correction using group as a covariate is shown with confidence intervals of the corrected data. For comparison, least square mean estimates (R package lsmeans) are used in Figure 1(e) to illustrate more appropriate confidence intervals that incorporate the uncertainties of the batch effect estimates.

## 3. Results

### 3.1. A simple sanity check

**Fig. 2.**
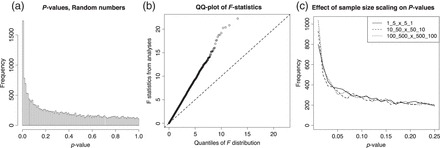
This is a sanity check where the use of ComBat with a covariate fails, adapted from the user guide in the sva package. Real data are substituted with random numbers from a normal distribution, but the batch–group design is retained. ComBat is applied, followed by an *F*-test. (a) *p*-Value distribution; (b) QQ plot of the *F*-statistics; (c) *p*-value distributions for 3 equally unbalanced random number experiments with different sample sizes, 12 120 and 1200 samples from two study groups with a 1:5 and 5:1 distribution in two batches, respectively. A random batch effect is added for 10% of the 20 000 genes. This example is not from the sva package.

The undesired consequences of preserving group effects when correcting for batch effect is readily illustrated with a sanity check using random numbers. The documentation accompanying the sva library (v3.8.0) has an executable example demonstrating how to adjust a data set with ComBat, followed by an *F*-test. Replacing the real data with random numbers from a standard normal distribution, $\textit {N}(0,1)$, but otherwise following the instructions, will generate the *p*-value distribution shown in Figure 2(a).

As can be seen from the QQ plot in Figure 2(b), the main effect of the procedure is to inflate the F-statistic by a factor. The size of this factor depends on how unbalanced the group–batch design is, rather than on the sample size in itself. Thus, increasing the sample size will not reduce the problem as shown in Figure 2(c).

If the number of samples is increased and there are no actual batch effects present, the empirical Bayes estimates used by ComBat will shrink the batch effect estimates and thus moderate the batch adjustments. However, if batch differences are added that are not constant across all genes, the problem remains even as the samples size increases.

### 3.2. Explanation for the simple two-group comparison

To explain more clearly what is happening, and to quantify the size of the problem, we may consider the simple case of estimating the difference $\Delta \beta =\beta _A-\beta _B$ between two groups, $A$ and $B$, when there are $m$ batches with batch $i=1,\ldots ,m$ containing $n_{iA}$ and $n_{iB}$ samples from each of the two groups.

#### 3.2.1. Group comparison from two-way ANOVA

If we estimate the group difference within batch $i$, we get

(3.1) \begin{equation*} \Delta\hat{\beta}_i=\bar{Y}_{iA}-\bar{Y}_{iB}\sim{\rm N}\left(\Delta\beta,\frac{\sigma^2}{\nu_i}\right) \quad{\rm where}\ \bar{Y}_{ij}=\frac{\sum_{r=1}^{n_{ij}}Y_{ijr}}{n_{ij}},\quad \nu_i=\frac{1}{{1}/{n_{iA}}+{1}/{n_{iB}}}, \end{equation*}

from which we may express the overall estimate of $\Delta \beta $

(3.2) \begin{equation*} \Delta\hat{\beta}=\frac{\sum_{i=1}^m \nu_i \Delta\hat{\beta}_i}{\nu} \sim{\rm N}\left(\Delta\beta,\frac{\sigma^2}{\nu}\right) \quad{\rm where}\ \nu=\sum_{i=1}^m\nu_i. \end{equation*}

This is the same as would be obtained from a two-way ANOVA analysis.

#### 3.2.2. Group comparison from one-way ANOVA after batch adjustment

If batch and group effects are estimated using a two-way ANOVA, the estimate $\Delta \hat {\beta }$ will be as stated above, and so the estimated group difference is unaffected. The batch effects are then removed, and the estimated group differences retained, leaving the estimated $\Delta \hat {\beta }$ unchanged by the batch adjustment.

However, if this batch-adjusted data set is analysed without considering batch effects, the variance of $\Delta \hat {\beta }$ will be computed under the assumption that it is derived from a comparison of $n_A=\sum _{i=1}^m n_{\textit {iA}}$ versus $n_{\!B}=\sum _{i=1}^m n_{\textit {iB}}$ samples, and thus satisfy $\Delta \hat {\beta }\sim {\rm N}(\Delta \beta ,\sigma ^2/\nu _0)$ where $1/\nu _0=1/n_A+1/n_B$. It follows from Jensen's inequality that $\nu _0\ge \nu $, with equality if and only if the ratios $n_{iA}:n_{iB}=n_A:n_B$ for all batches $i=1,\ldots ,m$.

In effect, $\nu $ represents the effective sample size in the unbalanced group–batch design, while $\nu _0$ represents the nominal sample size when batches are ignored. The ratio $\nu /\nu _0\le 1$ indicates to what extent the two-step procedure leads to underestimates of the random variability, and hence false or over-confidence in group differences.

### 3.3. Distribution of $F$-statistic in the general case

A one-way ANOVA checking for group differences between $M$ study groups in a data set of size $n$, will assume that the $F$-statistic follows an ${F}_{M-1,n-M}$ distribution as group differences have $M-1$ degrees of freedom, while there are $n-M$ degrees of freedom used to estimate the variance within groups.

When the $n$ observations are made in $m$ batches, with $n_{ij}$ samples in batch $i$ and group $j$, as in (2.1), and a two-way ANOVA is used to estimate and remove the batch effects, the batch-adjusted values are no longer independent. If a one-way ANOVA comparing study groups is run on the batch-adjusted data, the distribution of the $F$ statistic becomes

(3.3) \begin{equation*} F\sim\frac{\sum_{i=1}^{M-1}\lambda_i\chi^2_{1}}{\chi^2_{n-M-m+1}} \approx\frac{\tilde q\tilde\sigma^2}{(M-1)\sigma^2}\cdot\left(1+\frac{m-1}{n-M-m+1}\right)\cdot{\rm F}_{\tilde q,n-M-m+1} \end{equation*}

where the computation of $\lambda _i$, effective degrees of freedom $\tilde q$, and $\tilde \sigma ^2$ are derived in the Supplementary Material, and they depend on the batch–group design, not on the sample size. These satisfy $\tilde q\tilde \sigma ^2\ge $$(M-1)\sigma ^2$ and $\tilde q\le M-1$ with equalities if and only if the groups are evenly represented in all batches.

Even as sample size increases, assuming unchanged distribution of groups between batches, the factor $\tilde q\tilde \sigma ^2/(M-1)\sigma ^2$ will remain, consistently biasing the $F$ statistic towards higher values, as will the reduced degree of freedom $\tilde q$, which will increase the variance of the $F$ statistic.

### 3.4. Examples of undesired consequences

The extent to which batch adjustment with group differences retained will confound subsequent analyses depends on the batch–group balance. We have reanalyzed two cases with varying degree of unbalance.

#### 3.4.1. Experiment 1

**Fig. 3.**
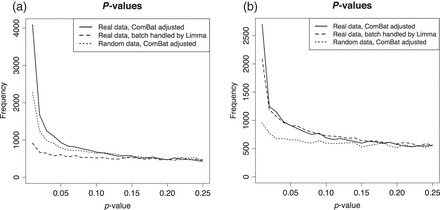
Three analyses of two published data sets where batch effects were adjusted for with ComBat. First, analyzed as described on the real data using ComBat. Secondly, with ComBat, but with random numbers instead of real data. Thirdly, instead of ComBat, analyses of the real data with limma blocking by batch. (a) Reanalysis of Towfic *and others* (2014), glatiramer acetate vs. generic, and (b) reanalysis of “Data set 2” (Johnson *and others*, 2007), TAL1 inhibition vs. control.

In an experiment described in Towfic *and others* (2014), the effect of Copaxone (glatiramer acetate, a medicine for multiple sclerosis, produced by Teva Pharmaceuticals) was compared with the effect of Glatimer, a generic version of the drug produced by Natco Pharma. Cells were treated with Copaxone (34 samples), Glatimer (11 samples), or one of 14 other treatments (60 samples), and mRNA expression was measured using microarrays. A batch effect correlating to the chip (Illumina WG-6_V2, six samples per chip, 17 chips in total) was observed and adjusted for with ComBat using treatment as a covariate. Batch-adjusted data were then tested for differentially expressed genes, yielding hundreds of differentially expressed probes (Table S5, Towfic *and others*, 2014) using a 5% FDR threshold. Unfortunately, the batch–treatment design was highly unbalanced, with several batches having only one of the main treatments of interest. We re-analyzed the cited data (GEO: GSE40566) as described, detecting 2011 differentially expressed genes at 5% FDR. When, instead of batch adjusting using ComBat, we blocked for batch effect in limma (Smyth, 2004), only 11 differentially expressed genes were detected at ${\rm FDR}<0.05$. The sanity check using random numbers, as described in Section 3.1, was also carried out. The distribution of *p*-values for different settings are shown in Figure 3(a).

It should be noted that the original analyses by Towfic *and others* (2014) differed from our re-analysis in one critical manner which was clarified upon contacting the authors. Their analyses were based on a different preprocessing of the microarray data (GEO: GSE61901), and included two technical replicates per biological sample corresponding to the two different slots of the bead-microarray. The inclusion of technical replicates as separate observations has a strong influence on the results regardless of the batch adjustment. For analyses where the two slots have been combined to provide one set of expression values per sample, they refer the reader to Bakshi *and others* (2013) in which Partek was used for batch adjustment.

#### 3.4.2. Experiment 2

The supporting information of the original ComBat article (Johnson *and others*, 2007) demonstrates the method on cells inhibited for the expression of the TAL1 gene compared to controls on a microarray platform (denoted “Data set 2”). The experiment consists of 30 samples in 3 batches: the number of treatment/control samples are batch 1: 6/2, batch 2: 3/4, and batch 3: 9/6. ComBat was applied followed by a *T*-test, to identify differentially expressed genes. First, we reproduced their analysis, including the adjustment by ComBat, but using limma instead of the *T*-test, resulting in 1003 probes ($q<0.05$). Then, we analyzed their data without batch adjustment in ComBat, but blocking for batch in limma, resulting in 377 probes ($q<0.05$). In addition, the sanity check using random numbers was performed. The distribution of *p*-values for different settings are shown in Figure 3(b). In contrast to the results obtained for Towfic *and others* (2014) (Figure 3(a)), the *p*-value distributions for the alternative analysis do not indicate a huge difference. Nevertheless, the *p*-values may still be somewhat deflated in the ComBat adjusted analysis.

## 4. Discussion

The use of study group, or other form of outcome, as a covariate when estimating and removing batch effects is problematic if the data are treated as “batch effect free” in subsequent analyses. When the group–batch distribution is unbalanced, i.e. where batches do not have the same composition of groups, this will lead to deflated estimates of the estimation errors, and over-confidence in the results. The problem is essentially independent of sample size as illustrated in Figure 3(c).

The size and impact of the problem will depend greatly on how unbalanced the group–batch distribution is: if it is only moderately unbalanced, it need not be a concern, whereas in heavily unbalanced cases it may have a huge influence. The impact is also more likely to be notable when used to analyze a large number of features, e.g. a large set of genes, followed by multiple testing corrections such as using the FDR, as the effect is more pronounced for more extreme values.

### 4.1. Motivation for this warning

Our knowledge of the problem discussed in this article came through a typical use case when trying to adjust for batch effects in an unbalanced data set using ComBat. Upon realizing that the confidence in the estimated group differences was exaggerated, we searched the literature for a better understanding of correct use and the potential limitations of ComBat. At the time, the authors of ComBat and the sva package recommended including study group as a covariate as we had done: the supporting documentation of sva has now been changed. Studies investigating batch effects were mostly recommending ComBat without much concern for potential limitations (Kupfer *and others*, 2012; Kitchen *and others*, 2011; Chen *and others*, 2011). A brief inquiry into some of the articles citing ComBat (466 in Web of Science) revealed few reported problems, although their method descriptions regarding ComBat were mostly sparse, limited to one or two sentences. Some used ComBat with covariates, some without, but we did not find any that addressed the potential problems related to use of covariates. However, the inability of batch adjustment methods in dealing with unbalanced designs has been noted (Buhule *and others*, 2014).

A further indication of the carefree use of the procedure was the frequent omission of program parameters that were applied, i.e. batch labels or whether group labels were supplied as covariates. Often, no effort was undertaken in order to substantiate the existence of batch effects, beyond stating the presence of batches. The incorporation of ComBat and other batch adjustment methods into various analysis tools (see Supplementary Material) may make them more accessible, but their use less transparent.

As we looked into the problems of batch-adjusting unbalanced data sets, we realized that these are shared by other batch adjustment methods: indeed, the empirical Bayes approach used by ComBat may dampen the batch adjustments and thereby reduce the problem slightly relative to a more direct two-way ANOVA approach. Although some of the tools warn against performing subsequent analyses on batch-adjusted data, these warnings are often somewhat vague and seem to be primarily concerned about the degrees of freedom in the data set. Batch adjustment will reduce the degrees of freedom in the data which may influence the distribution of the test statistics in subsequent analyses, e.g. the variance of the $F$-statistics from ANOVA. For small data sets or where there are many small batches, this may be a problem, while it would be less of a concern when there are a fair number of samples in each batch. However, we find that the main problem is a systematic inflation of the $F$-statistic by a factor, induced as estimation errors during the batch adjustments are applied across the data, which can induce apparent group differences in the adjusted data set even in the absence of batch effects.

We are concerned, in light of our findings, that many published results from batch-adjusted data using the study group as a covariate may be unreliable. Furthermore, the frequent lack of proper method description accompanying published results makes it hard to judge if they are affected or not. We hope that our warning will help caution the scientific community against this particular approach.

### 4.2. Practical advice

The main advice, particularly when batch effects are significant, is to ensure a balanced design in which study groups are evenly distributed across batches.

For an investigator facing an unbalanced data set with batch effects, our primary advice would be to account for batch in the statistical analysis. If this is not possible, batch adjustment using outcome as a covariate should only be performed with great caution, and the batch-adjusted data should not be trusted to be “batch effect free”, even when a diagnostic tool might claim so. Comparing results from batch-adjusted data with and without covariates may indicate to what extent the outcome depends on the mode of batch adjustment.

Alternatively, one may treat the results more like an ordered list of candidates, with the most likely true positives on top, de-emphasizing the somewhat deflated $p$-values. This would, however, be hypothesis-generating, rather than hypothesis-testing. While investigators should always assess the extent to which findings make biological or clinical sense, this is particularly true when the statistical assessment may be unreliable.

Knowing that adjustment for batch effects while preserving the group difference may lead to varying degree of false results, to what extent can an investigator trust published results where such a method has been applied? Essentially, when the batch–group configuration is balanced, group differences are not affected by batch effects and results should be reliable. For unbalanced designs, however, where batch effects may act as a confounder, the most rigorous path would be a reanalysis which accounts for batch effect, rather than relying on the two-step approach. However, such a reanalysis may be infeasible or impractical, depending on the availability of raw data and proper method description, the ability of analysis tools to handle batch effects, and the necessary statistics and bioinformatics resources. In any case, to reach a reliable result, batch effects need to be handled in some way or another.

We have provided explicit expressions both for the two-group comparison and the adjusted $F$ distribution for the more general linear model. Although it is possible to use these formulas to correct the results, this is essentially the same as running two-way ANOVA or a general linear model including batch, which in most cases would be an easier, safer, and more general solution, and more powerful as the former approach does not fully exploit the degrees of freedom of the model. However, the expressions derived in Sections 3.2 and 3.3 may still be used to assess the reliability of results based on batch adjusted data, and we provide some support for performing these calculations at the Additional analyses page at GitHub The main concern would be batches where either of the groups of interest are missing or strongly under-represented, which would contribute to the nominal sample size, but not to the effective sample size. In Section 3.2, the ratio $\nu _0/\nu \ge 1$ indicates by how much the effective sample size is overestimated when comparing two groups. If the $\nu _0/\nu $ ratio is close to 1, results should still be reliable, while a ratio much larger than 1 will indicate exaggerated confidence. A similar computation, summarized in Section 3.3, although more complicated, can be made for multiple groups or general linear models. If the behavior of the test statistic is well understood, as the $F$-statistic in ANOVA, one may adjust the test statistic accordingly. However, this will require deep insight into the statistical behavior of the model. Down-sampling to the effective samples size, i.e. to a $\nu /\nu _0$ portion of the samples, may also be done to see if results persist. However, none of these solutions are ideal.

Finally, we would like to emphasize the importance of proper description of how data have been prepared for analysis, and what corrections and adjustments have been made (Sandve *and others*, 2013). In cases where data preparation is performed prior to, and separate from, the data analysis, as is now often the case, this is of particular importance, as artifacts may be introduced in the data preparation which could influence the reliability of downstream analyses. When assessing published findings, we frequently found it hard to determine how batch adjustments had been done, and occasionally suspected that the study group had been used as a covariate without this being stated. Science not only depends on determining sound methods and finding reliable results, but also on communicating sufficient detail to allow readers to assess the extent to which findings can be trusted. Failing to do so may cause methodological problems to pass undetected, but also to cast doubt on sound results. An increased focus on making research reproducible is therefore of great importance.

## Supplementary material

Supplementary material is available here.

## Reproducible research

Data and scripts used to generate these are available at https://github.com/ous-uio-bioinfo-core/batch-adjust-warning-figures.git. Additional analyses and resources may be found at https://github.com/ous-uio-bioinfo-core/batch-adjust-warning-reports.git.

## Funding

This work was supported by the EUROCAN platform (VN, FP7/2007-2013, grant 260791) and the MetAction project (EAR). Funding to pay the Open Access publication charges for this article was provided by the Department of Tumor Biology, Institute for Cancer Research, Oslo University Hospital.

## Supplementary Material

Supplementary Data
