# Supplementary material for: Methods that remove batch effects while retaining group differences may lead to exaggerated confidence in downstream analyses
Source: Biostatistics. 2015 Aug 13;17(1):29–39. doi: 10.1093/biostatistics/kxv027 (PMC4679072; doi:10.1093/biostatistics/kxv027)
Supplement: Supplementary Data [file kxv027supp_data.pdf]

Supplementary information

Methods that remove batch effects while retaining group differences may lead to exaggerated confidence in downstream analyses

VEGARD NYGAARD, EINAR ANDREAS RØDLAND

Department of Tumor Biology, Institute for Cancer Research, Oslo University Hospital HF - Radiumhospitalet, Montebello, 0310 Oslo, Norway

EIVIND HOVIG

Department of Tumor Biology, Institute for Cancer Research, Oslo University Hospital HF - Radiumhospitalet, Montebello, 0310 Oslo, Norway

Institute of Cancer Genetics and Informatics, Oslo University Hospital HF - Radiumhospitalet, Montebello, 0310 Oslo, Norway

Department of Informatics, University of Oslo, 0316 OSLO Norway

ehovig@ifi.uio.no

1. Distribution of error terms after two-way ANOVA batch adjustment

1.1 *Statistical model*

We assume a general linear model with intercept or more general covariates (α), study groups or study parameters of interest (β), and batch effects or other covariates to adjust for (γ):

$$Y = \alpha A + \beta B + \gamma C + \epsilon \tag{1.1}$$

where $Y = [Y_1, \ldots, Y_n]$ are the observable random variables, parameters α, β, and γ are p, q, and r vectors with design matrices A, B, and C, and $\epsilon = [\epsilon_1, \ldots, \epsilon_n] \sim \mathrm{N}(0, \sigma^2 I_n)$ where I_n is the $n \times n$ identity matrix. We also assume that the full design matrix, combining A, B, and C, has full rank $p + q + r$: i.e., that the covariates are linearly independent.

In general, we wish to assess the explanatory power of B when including C as batch effects and A as additional covariates. Often, $p = 1$ with α the intercept term, but this formulation allows general covariates to be included in the analyses. The covariates of B which are subject to testing are more commonly specified as contrasts on the full design matrix combining A, B, and C, but in our case this splitting is more convenient.

Least squares estimates for the parameters can be obtained through

$$[\hat{\alpha}|\hat{\beta}|\hat{\gamma}] = YX^t(XX^t)^{-1} = [\alpha|\beta|\gamma] + \epsilon X^t(XX^t)^{-1} \quad \text{where} \quad X = \begin{bmatrix} A \\ B \\ C \end{bmatrix}. \tag{1.2}$$

1.2 *Elimination of covariates A*

We can eliminate A from the model, simultaneously removing p degrees of freedom. One way of doing this is to find an $n \times n$ rotation matrix R, i.e. with $RR^t = I$, so that $AR = [0|A']$ with A' a $p \times p$ invertible matrix. Applying this rotation to all elements, we split the n dimensions into $(n-p)+p$: $YR = [Y_0|Y']$, $BR = [B_0|B']$, $CR = [C_0|C']$, and $\epsilon V = [\epsilon_0|\epsilon'] \sim \mathrm{N}(0, \sigma^2 I_n)$. We may then eliminate the degrees of freedom used to estimate α from the model yielding

$$Y_0 = \beta_0 B_0 + \gamma_0 C_0 + \epsilon_0 \tag{1.3}$$

and Y_0 and ϵ_0 are now $n-p$ vectors. If α is the intercept term, this corresponds to centering all variables and covariates, but the rotation also eliminates the corresponding degree of freedom. Parameter estimates are now given by

$$[\hat{\beta}_0|\hat{\gamma}_0] = [\beta_0|\gamma_0] + \epsilon X_0^t(X_0X_0^t)^{-1} \quad \text{where} \quad X_0 = \begin{bmatrix} B_0 \\ C_0 \end{bmatrix}. \tag{1.4}$$

Eliminating the covariates in this manner is strictly not required. We could have done all computations on the full model, but this elimination makes the computations a little simpler to deal with and present.

1.3 *F statistic for model without batch effects*

If batch effects are not included in the model, i.e. $r = 0$, we get the common linear model in which we get a decomposition $Y_0 = \hat{\beta}_0 B_0 + \hat{\epsilon}_0$ with $\hat{\beta}_0 = Y_0 B_0^t (B_0 B_0^t)^{-1}$, where $|\hat{\epsilon}|^2 \sim \sigma^2 \chi^2_{n-p-q}$ and $|(\hat{\beta}_0 - \beta_0)B_0|^2 \sim \sigma^2 \chi^2_q$, yielding the common F-statistic

$$F = \frac{|(\hat{\beta}_0 - \beta_0)B_0|^2/q}{|\hat{\epsilon}_0|^2/(n-p-q)} \sim \mathrm{F}_{q,n-p-q}. \tag{1.5}$$

If we believe our data are "batch effect free", and therefore do not include batch in the model, this is the distribution of the F-statistic we would assume. Hence, this will be the assumed distribution when batch adjusted data are analysed using a linear model without batch effects included.

1.4 *Distribution of F statistic after batch adjustment*

We define batch adjusted data $\tilde{Y} = Y - \hat{\gamma}C$ using the estimated batch effect $\hat{\gamma}$ from the full model using (1.2). After elimination of covariates A, the batch adjusted data becomes $\tilde{Y}_0 = Y_0 - \hat{\gamma}_0 C_0$ where parameter estimates $[\hat{\beta}_0|\hat{\gamma}_0]$ are given by (1.4). The linear decomposition $Y_0 = \hat{\beta}_0 B_0 + \hat{\gamma}_0 C_0 + \hat{\epsilon}_0$ has $|\hat{\epsilon}_0|^2 \sim \sigma^2 \chi^2_{n-p-q-r}$ and $|(\hat{\beta}_0 - \beta_0)B_0 + (\hat{\gamma}_0 - \gamma_0)C_0|^2 \sim \sigma^2 \chi^2_{q+r}$, and so the variance of the error term is the same in the batch adjusted case $\tilde{Y}_0 = \hat{\beta}_0 B_0 + \hat{\epsilon}_0$. However, the sum of squares $|(\hat{\beta}_0 - \beta_0)B_0|^2$ explained by B is less easily expressed.

For convenience, we define the projection ρ_W to the $n-k$-hyperplane perpendicular to the k-hyperplane spanned by the rows of W for any non-degenerate $k \times n$ matrix with $k \leqslant n$,

$$\rho_W = I - W^t(WW^t)^{-1}W \quad \text{and} \quad \rho_{U,V} = \rho_W \text{ where } W = \begin{bmatrix} U \\ V \end{bmatrix}, \tag{1.6}$$

through which solutions will be more easily expressed.

Determining $\hat{\beta}_0 B_0$, using $[\hat{\beta}_0|\hat{\gamma}_0] = Y_0 X_0^t (X_0 X_0^t)^{-1}$ and

$$X_0^t(X_0X_0^t)^{-1}\begin{bmatrix} B_0 \\ 0 \end{bmatrix} = [B_0^t|C_0^t]\begin{bmatrix} P \\ -(C_0C_0^t)^{-1}C_0B_0^tP \end{bmatrix} B_0 = \rho_{C_0}B_0^tPB_0 \tag{1.7}$$

with $P = (B_0\rho_{C_0}B_0^t)^{-1}$, yields

$$(\hat{\beta}_0 - \beta_0)B_0 = \epsilon_0\rho_{C_0}B_0^tPB_0 \sim \mathrm{N}(0, \sigma^2 L) \quad \text{where} \quad L = B_0^tPB_0 \tag{1.8}$$

since $\epsilon_0 \sim \mathrm{N}(0, \sigma^2 I)$. Let $\lambda_1, \ldots, \lambda_q \geqslant 1$ be the non-zero eigenvalues of L: this inequality holds since L is a non-degenerate symmetric rank q matrix, and has the same eigenvalues as $PB_0B_0^t$ the inverse of which, $(B_0B_0^t)^{-1}B_0\rho_{C_0}B_0^t$, has eigenvalues in $(0,1)$ (not 0 since B_0 and C_0 are linearly independent). Then, by decomposing the variation along the eigenvectors,

$$|(\hat{\beta}_0 - \beta_0)B_0|^2 = \sigma^2\sum_{i=1}^{q}\lambda_i S_i \quad \text{where} \quad S_1, \ldots, S_q \sim \chi_1^2. \tag{1.9}$$

Using Satterthwaite's approximation, which matches the first two momenta, we get

$$|(\hat{\beta}_0 - \beta_0)B_0|^2 \sim \sigma^2\sum_{i=1}^{q}\lambda_i\chi_1^2 \approx \tilde{\sigma}^2\chi_{\tilde{q}}^2 \tag{1.10}$$

where

$$\frac{\tilde{q}\tilde{\sigma}^2}{\sigma^2} = \sum_{i=1}^{q}\lambda_i = \mathrm{tr}(L) \quad \text{and} \quad \frac{\tilde{q}\tilde{\sigma}^4}{\sigma^4} = \sum_{i=1}^{q}\lambda_i^2 = \mathrm{tr}(L^2). \tag{1.11}$$

From $\lambda_i \geqslant 1$ follows that $\tilde{\sigma}^2 \geqslant \sigma^2$, $\tilde{q}\tilde{\sigma}^2 \geqslant q\sigma^2$, and $\tilde{q} \leqslant q$. Equality happens when all $\lambda_i = 1$, which occurs when B_0 and C_0 are orthogonal, i.e. $B_0C_0^t = 0$.

The accuracy of Satterthwaite's approximation depends on the variability of the eigenvalues λ_i. If these are all identical, the approximation is exact. However, if the eigenvalues vary greatly, the extreme upper tail of the distribution will be more heavily influenced by the larger eigenvalues, and thus have a longer upper tail, than the approximation takes into account.

1.5 *Reformulation in terms of the original design matrices*

We would like to express $\tilde{\sigma}^2$ and $\tilde{q}$ terms of the original design matrices, i.e. before elimination of covariates. The rotated design matrices were defined as $B_0 = BR_0$, etc., using the rotation $R = [R_0|R']$ so that $AR_0 = 0$ while AR' is non-singular. Hence, it follows that $R_0R_0^t = \rho_A$. This allows us to express

$$L = R_0^tB^tPBR_0 \quad \text{where} \quad P = (BR_0\rho_{C_0}R_0^tB^t)^{-1} = (B\rho_{A,C}B^t)^{-1}. \tag{1.12}$$

We can then compute $\tilde{q}$ and $\tilde{\sigma}^2$ from

$$\frac{\tilde{q}\tilde{\sigma}^2}{\sigma^2} = \mathrm{tr}(M), \quad \text{and} \quad \frac{\tilde{q}\tilde{\sigma}^4}{\sigma^4} = \mathrm{tr}(M^2) \quad \text{with} \quad M = B\rho_A B^t (B\rho_{A,C} B^t)^{-1} \tag{1.13}$$

since $M = BR_0R_0^tB^tP$ and $L = R_0^tB^tPBR_0$ have the same non-zero eigenvalues. Notice that if B is orthogonal to A, which can be achieved by replacing B with $B - VA$ for some matrix V, then $B\rho_A = B$. If C is also made orthogonal to A, then $\rho_{A,C}$ can be replaced by ρ_C.

1.6 *Comparison to running full ANOVA or linear model*

If the full model, including batch effects, is analysed using a traditional ANOVA or linear model approach, the result will be an F-statistic with distribution $F \sim \mathrm{F}_{q,n-p-q-r}$: the q degrees of freedom for measuring the effect contribute evenly to the statistic.

If batch-adjusted data are analysed without including covariates, the q degrees of freedom end up scaled up by factors $\lambda_1, \ldots, \lambda_q$. The corrected F-distribution can account for this. However, if the λ_i eigenvalues vary, some degrees of freedom will contribute more to the F-statistic than others, which makes for inefficient use of the data, and results in a reduction in the effective degrees of freedom to $\tilde{q}$: the F-statistic becomes more variable than that from the common linear modelling approach which exploited the full q degrees of freedom.

Thus, although an appropriate distribution of the F-statistic can be found for the two-step approach, there will still be some loss of power.

1.7 *Implementation in R*

In order to demonstrate the computations, we provide the R script `theory/F-distribution.r` at `https://github.com/ous-uio-bioinfo-core/batch-adjust-warning-reports.git`.

2. Methods for batch adjustment

2.1 *Methods similar to ComBat*

Though our work was originally focused on ComBat, we realise that other methods are also available which remove batch effects in much the same way and thus have the same problems. Below is a list of the methods encountered along with a brief description.

2.1.1 *Partek* The commercial software Partek Genomics Suite is commonly used software for analysing genomic data. Included in it is a Batch Remover tool which, based on the user guide (obtained from Partek support), seems to estimate the batch and group effects using an ANOVA model and then remove the estimated batch effects. It is clear from the user guide that the main purpose of this tool is for visualization, and if further statistical tests are performed on the data set, the batch factor must still be included.

2.1.2 *removeBatchEffect* The method `removeBatchEffect` found in the popular `limma` (Smyth and Speed, 2003) package adjusts for batch effects using an ANOVA model with batch and groups included. The methods help description states that its use is for visualization and not for further linear modelling.

2.1.3 *ber* The recent R package `ber` (Giordan, 2013) uses, in addition to ANOVA, bagging to better estimate the batch effects. Inclusion of group labels for which the effects should be preserved seems to be its recommended use. So far 4 citations are listed in google scholar

2.2 *Applications and pipelines that use ComBat*

The inclusion of ComBat in numerous pipelines makes its use easier, but may also make it more difficult to identify the potential problems we discuss. We found several pipelines or libraries with ComBat included, some are soft wrappers, while others have a more worrisome implementation. The short descriptions below are made from a shallow review of the article, tutorial or code. We have not tried them out.

2.2.1 *ChAMP* ChAMP (Morris *and others*, 2014, version 1.2.8) is a pipeline for analysing the llumina Infinium HumanMethylation450 BeadChip. Batch effect adjustment is optional with a modified version of ComBat specifically implemented to use the BeadChip, which consist of 12 samples, as batch. From the tutorial example, no parameters are passed to their ComBat implementation. However, inspection of the code shows that sample information entered earlier will automatically be used as covariates. This recent tool has already 15 citations in google scholar.

2.2.2 *intCor* The `intCor` package (Fernández-Albert *and others*, 2014, version 1.03) may be used to analyse data from liquid chromatography coupled to mass spectrometry experiments. ComBat correction is optional and it seems that group assignments entered earlier in the pipeline is automatically used as covariates. This new pipeline has not many users so far.

2.2.3 *AltAnalyze* AltAnalyze (Emig *and others*, 2010, version 2.0.8) is a tool for analysing alternative splicing. ComBat as an option was included in 2013. The user guide states that prior entered group assignments will be used as covariates. ComBat is here implemented in Python rather than R. The tool has 59 citations in Google scholar, most prior to the inclusion of ComBat.

2.2.4 *inSilicoMerging* The R package `inSilicoMerging` (Taminau *and others*, 2012, version 1.8.7) combines public available microarray gene expression data. The implementation of ComBat is presently without covariates as an option. It has 12 citations according to google scholar.

2.2.5 *GenePattern* GenePattern (Reich *and others*, 2006, version 3.9.0) is a popular platform for analysing gene expression data. It incorporates a lot of tools including ComBat. The implementation is a soft wrapper around ComBat, and the inclusion of covariates is handled similar.

2.2.6 *SCAN.UPC* SCAN.UPC (Piccolo *and others*, 2013, version 2.6.3) is a microarray normalization method d to facilitate personalized-medicine workflows. It includes a soft wrapper around ComBat, which seems to be made out of convince and operates as the original. Currently it has 7 citations in google scholar.

2.2.7 *TCGA* The Cancer Genome Atlas generates and shares lots of data, some with batch effects. Several batch adjustment tools are implemented, including ComBat. However, it seems that the inclusion of covariates is not an option.

References

Emig, D., Salomonis, N., Baumbach, J., Lengauer, T., Conklin, B. R. and Albrecht, M. (2010, July). AltAnalyze and DomainGraph: analyzing and visualizing exon expression data. *Nucleic acids research* **38**(Web Server issue), W755–62.

Fernández-Albert, F., Llorach, R., Garcia-Aloy, M., Ziyatdinov, A., Andres-Lacueva, C. and Perera, A. (2014, October). Intensity drift removal in LC/MS metabolomics by common variance compensation. *Bioinformatics (Oxford, England)* **30**(20), 2899–905.

Giordan, M. (2013, February). A Two-Stage Procedure for the Removal of Batch Effects in Microarray Studies. *Statistics in Biosciences* **6**(1), 73–84.

Morris, T. J., Butcher, L. M., Feber, A., Teschendorff, A. E., Chakravarthy, A. R., Wojdacz, T. K. and Beck, S. (2014, March). ChAMP: 450k Chip Analysis Methylation Pipeline. *Bioinformatics (Oxford, England)* **30**(3), 428–30.

Piccolo, S. R., Withers, M. R., Francis, O. E., Bild, A. H. and Johnson, W. E. (2013, October). Multiplatform single-sample estimates of transcriptional activation. *Proceedings of the National Academy of Sciences of the United States of America* **110**(44), 17778–83.

Reich, M., Liefeld, T., Gould, J., Lerner, J., Tamayo, P. and Mesirov, J. P. (2006, May). GenePattern 2.0. *Nature genetics* **38**(5), 500–1.

Smyth, G. K. and Speed, T. (2003, December). Normalization of cDNA microarray data. *Methods* **31**(4), 265–273.

Taminau, J., Meganck, S., Lazar, C., Steenhoff, D., Coletta, A., Molter, C., Duque, R., de Schaetzen, V., Weiss Solís, D. Y., Bersini, H. *and others*. (2012, January). Unlocking the potential of publicly available microarray data using inSilicoDb and inSilicoMerging R/Bioconductor packages. *BMC bioinformatics* **13**, 335.
